# Repetitive Overuse Injury Causes Entheseal Damage and Palmar Muscle Fibrosis in Older Rats

**DOI:** 10.3390/ijms252413546

**Published:** 2024-12-18

**Authors:** Parth R. Patel, Istvan P. Tamas, Megan Van Der Bas, Abby Kegg, Brendan A. Hilliard, Alex G. Lambi, Steven N. Popoff, Mary F. Barbe

**Affiliations:** 1 Aging + Cardiovascular Discovery Center, Lewis Katz School of Medicine at Temple University, Philadelphia, PA 19140, USA; ppatel2015@temple.edu (P.R.P.); istvan.tamas@temple.edu (I.P.T.); megan.vanderbas@temple.edu (M.V.D.B.); abby.kegg@temple.edu (A.K.); brendan.hilliard@temple.edu (B.A.H.); 2 Plastic Surgery Section, New Mexico Veterans Administration Health Care System, Albuquerque, NM 87108, USA; alambi@salud.unm.edu; 3 Department of Surgery, The University of New Mexico School of Medicine, Albuquerque, NM 87106, USA; 4 Department of Biomedical Education and Data Science, Lewis Katz School of Medicine at Temple University, Philadelphia, PA 19140, USA; steven.popoff@temple.edu

**Keywords:** work-related musculoskeletal disorder, enthesis, cartilage, grip strength, TNFalpha, aging, fibrosis

## Abstract

Overuse injury is a frequent diagnosis in occupational medicine and athletics. Using an established model of upper extremity overuse, we sought to characterize changes occurring in the forepaws and forelimbs of mature female rats (14–18 months of age). Thirty-three rats underwent a 4-week shaping period, before performing a high-repetition low-force (HRLF) task for 12 weeks, with the results being compared to 32 mature controls. HRLF animals showed a reduced grip strength versus controls. ELISAs carried out in the HRLF rats, versus controls, showed elevated levels of IL1-α in tendons, IL1-α and TNF-α in distal bones/entheses, and TNF-α, MIP1-α/CCL3, and CINC-2/CXCL-3 in serum, as well as IL-6 in forelimb muscles and tendons, and IL-10 in serum. HRLF rats had elevated collagen deposition in the forepaw intrinsic muscles (i.e., fibrosis), entheseal microdamage, and articular cartilage degradation versus the control rats. CD68/ED1+ osteoclasts and single-nucleated cells were elevated in distal forelimb metaphyses of the HRLF animals, versus controls. Declines in grip strength correlated with muscle fibrosis, entheseal microdamage, articular cartilage damage, distal bone/enthesis IL1-α, and serum IL-6. These data demonstrate inflammatory and persistent degradative changes in the forearm/forepaw tissues of mature female animals exposed to prolonged repetitive tasks, changes with clinical relevance to work-related overuse injuries in mature human females.

## 1. Introduction

Overuse injuries are one of the most common forms of musculoskeletal disorders, affecting people of a wide range of ages and occupations. Those developed at the workplace, referred to as work-related musculoskeletal disorders (WRMSDs), often arise in patients with known risk factors such as highly repetitive tasks, poor ergonomics and awkward work positions, vibratory tasks, female gender, and advancing age [1]. In general, WRMSDs constitute a large percentage of disabilities and account for up to a third of missed workdays [2]. This leads to exorbitant annual health care expenditures and high lost productivity costs [3].

WRMSDs of the upper extremity, specifically the forearm, wrist, and hand, are common and often persist, causing continual disruptions to job performance, represented by a high number of missed work days, restricted work duties, and job transfers [4,5]. These disorders are associated with motor declines, discomfort and pain [6], and fibrotic changes in biopsied tissues [7,8]. WRMSDs are also associated with the development of tendinitis, epicondylitis, joint pathologies, Dupuytren’s, and more [9,10,11,12,13,14,15,16,17,18]. WRMSDs in these upper extremity regions were noted to affect 20,000 US individuals in 2020 alone, making up a significant portion of the reported total [4].

Additionally, the data on WRSMDs clearly show that these injuries become far more proportionally present in older work populations, such as those within age groups of 45–54 and 54–65 [2,4,19,20]. Along with an increased prevalence, work-related injuries in older worker are also associated with more time missed, higher expenses being incurred, and reoccurrence [20]. These associations between aging and WRMSDs are well represented across the globe, and contribute to increased costs to individuals, organizations, and countries [21]. Given the increases in longevity and the continued importance of aging workers to the workforce, it is recognized as a mutual priority to better support this population and address preventable injuries.

Our group has developed a volitional animal model of upper extremity overuse injuries in which rats perform a reaching lever-bar-pulling task. Motivated by a food reward, the rats learn to pull the lever at defined reach rates and target forces, which induce dose-dependent changes in inflammatory and degradative responses in young adult rats (3.5–6.5 months of age) [22]. Inflammatory responses were transient in young adult rats performing low- or moderate-force repetitive tasks, but higher and more sustained in young adult rats performing a high-force repetitive task [22]. In contrast, studies examining mature female rats (14–18 months of age) versus young adult female rats performing the same low-force repetitive task for 12 weeks (termed hereafter as HRLF rats) found enhanced inflammatory cytokine responses in the serum, forelimb and shoulder tendons, and forelimb bones of the mature HRLF rats compared to the young adult HRLF rats [23,24,25]. These findings are supportive of age-related elevations in inflammatory responses after injury, as well as baseline levels, known as “inflammaging”, which can drive tissue pathogenesis [26,27]. For example, enhanced tendon inflammation was associated with epitendon fibroplasia and entheseal microdamage in the forelimb flexor and supraspinatous tendons, respectively, in mature HRLF rats, changes that were absent in the young adult HRLF rats [24]. Furthermore, the forelimb bones of mature HRLF rats showed higher osteoclast numbers and activity, enhanced trabecular bone loss and degradation, and cortical bone thinning compared to young adult HRLF rats that, in contrast, displayed enhanced trabecular bone volume and bone formation [25].

Although not yet examined in mature HRLF rats, the muscle and fascial tissues of young adult rats also showed dose-dependent changes in fibrotic and degenerative responses, with a lower production of fibrosis-related proteins (e.g., collagen type 1) and less tissue degradation in young adult HRLF rats than young adult rats performing high-force repetitive tasks [22,28]. In the young adult rats, inflammation was a key underlying mechanism of task-induced tissue fibrosis and degradation [29,30], and each tissue response (inflammation, fibrosis, and degradation) contributed to functional declines [22,31]. Although we have reported elevated inflammatory cytokines in mature HRLF rats, we have yet to explore forearm and forepaw fibrogenic or degradation changes in these rats and if they correlate with functional declines, e.g., in grip strength, a functional biomarker that has been used as a biological marker of aging [32,33].

Entheses are dense connective tissues which attach tendons and ligaments to bone. Dense connective tissues that connect other tissues fall under the umbrella of fascia [34,35]. Given that the enthesis represents the dense connective tissue/fascial transition zone of the tendon to the epimysium, it is frequently subject to high stress levels and is thus vulnerable to overuse injury [36,37,38]. Studies using animal models describe entheseal changes with intense exercise or high-force repetitive tasks [31,39]. Articular cartilage serves a similar function to entheses in facilitating the transmission of load and withstanding compression [40,41]. Although articular cartilage is also a dense connective tissue, it is not considered a fascial tissue. We have observed disruption of cartilage structure in young adult female rats performing the intense HRHF task [29]. No studies to date have examined forelimb/forepaw entheseal and cartilage changes in mature animals performing low-force repetitive tasks.

Therefore, we expanded from our past studies to instead examine inflammatory and structural changes in forelimb muscles and tendons, forepaw muscles, distal forelimb bones with entheses, and articular cartilage in the distal forepaws and wrists (i.e., the muscle-tendon-bone interfaces) of mature rats (14–18 months of age) performing an HRLF task for 12 weeks, relative to age-matched control rats. We also sought to determine if such changes are associated with functional motor declines. We hypothesized that the mature rats performing the HRLF task would display higher inflammatory cytokine production, forepaw muscle fibrosis, and entheseal and cartilage microdamage, and that these changes would correlate with grip strength.

## 2. Results

### 2.1. Functional Performance over Time

**Figure 1:**
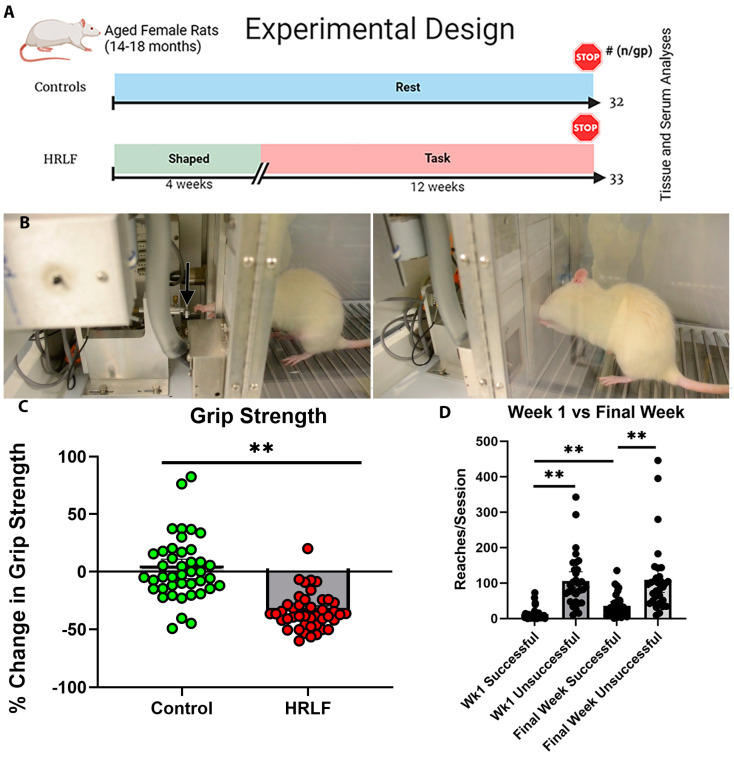
Design, reach performance, and grip strength. (**A**) Experimental design. Sample size per group (n/gp) is noted on the right. Rats were shaped over 4 weeks to learn the reaching and lever-pulling task. They then performed a high-repetition low-force task for 12 weeks (HRLF, 4 reaches/min target at 15% of maximum pulling force, 2 h/day, 3 days/week). Results were compared to age-matched controls. (**B**) Images of rat pulling on the lever bar (arrow) located 2.5 cm outside of an operant chamber at shoulder height. (**C**) A graph showing the percent change in grip strength from week 0 to week 12. (**D**) Graph showing reaches per session by the task rats on the y-axis. The x-axis shows the number of successful and unsuccessful reaches performed by task rats in weeks 1 and 12 (final week). These latter data could not be reported for the control rats as they did not perform the task. ** *p* < 0.01, compared between groups as shown.

We have previously shown a decrease in grip strength in mature HRLF rats [42]. Similarly, in this study, we observed a loss of grip strength from baseline levels (presented as percent change in grip strength) in 12-week mature HRLF rats (*p* < 0.01) compared to mature control rats (Figure 1C). Several of the HRLF rats tended to overreach (more than the target of 4 reach/min, 120 reaches/session) (Figure 1D). Although the target force for a food reward was 26.4 ± 1.32 cN, the rats pulled with a higher range [their maximum average force was 34.48 cN and the minimum average force was 24.63 cN. As a consequence, the HRLF animals also had more unsuccessful pulls than successful pulls in both tasks in week 1 and in the final week (week 12; *p* < 0.01; Figure 1D). There was a small yet significant increase in successful pulls when comparing the final week to the first (*p* < 0.01, Figure 1D), suggestive of learning. This latter data could not be reported for the control rats as they did not perform the task.

### 2.2. Several Pro-Inflammatory and Anti-Inflammatory Cytokines Changed in Tissues and Serum of Mature HRLF Rats

**Figure 2:**
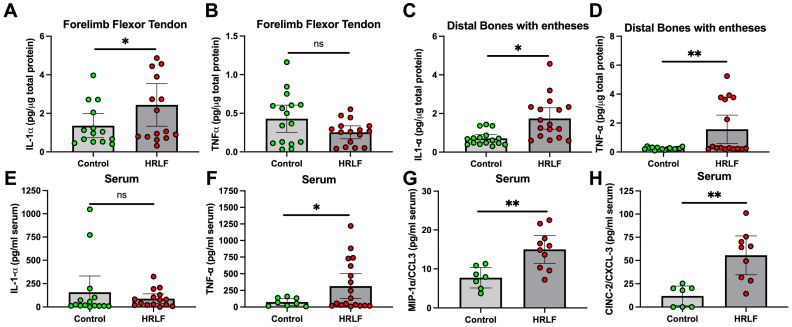
Pro-inflammatory cytokine levels detected using ELISA. (**A**,**B**) IL-1α and TNF-α levels in forelimb flexor tendons. (**C**,**D**) IL-1α and TNF-α levels in distal bone with entheses. (**E**–**H**) IL-1α, TNF-α, MIP1-α/CCL3, and CINC-2/CXCL-3 levels in serum. Musculoskeletal tissues were assayed in Control (*n* = 17–21) and HRLF (*n* = 18–19) animals; serum was assayed in Control (*n* = 7–15) and HRLF (*n* = 9–19) animals. * *p* < 0.05 and ** *p* < 0.01, compared between groups as shown; ns = not significant.

In an effort to understand the underlying causes of motor function declines, we examined key pro-inflammatory cytokines in the forelimb/forepaw musculoskeletal tissues and serum using ELISA. The forelimb flexor muscles of the forelimb showed no changes in interleukin 1 alpha (IL-1α) between the two groups [HRLF (0.09 ± 0.05 to 0.13 95% CI) versus Control (0.08 ± 0.06 to 0.09 95% CI) *p* = 0.86], interleukin 1 beta (IL-1beta) [HRLF (0.21 ± 0.04 to 0.23 95% CI) versus Control (0.20 ± 0.05 to 0.23 95% CI) *p* = 0.89], or tumor necrosis factor alpha (TNF-α) [HRLF (0.05 ± 0.04 to 0.05 95% CI) versus Control (0.05 ± 0.04 to 0.06 95% CI), *p* = 0.78] (Supplemental Figure S1A–C). In contrast, the flexor tendons in HRLF rats showed elevated IL-1α compared to the control rats (*p* < 0.05, Figure 2A), although there were no differences in TNF-α levels (Figure 2B). The distal forelimb bones with entheses in the HRLF rats showed elevated levels of IL-1α and TNF-α compared to the control rats (*p* < 0.05 and *p* < 0.01, Figure 2C and D, respectively). In the serum, there was no difference in IL-1α levels between the groups (Figure 2E). However, circulating levels of TNF-α, macrophage inflammatory protein 1 alpha/C-C motif chemokine ligand 3 (MIP1-α/CCL3), and cytokine-induced neutrophil chemoattractant 2/C-X-C motif ligand 3 (CINC-2/CXCL-3) were elevated in the HRLF rat serum compared to the control rats (*p* < 0.05, *p* < 0.01, and *p* < 0.01, respectively, Figure 2F–H).

**Figure 3:**
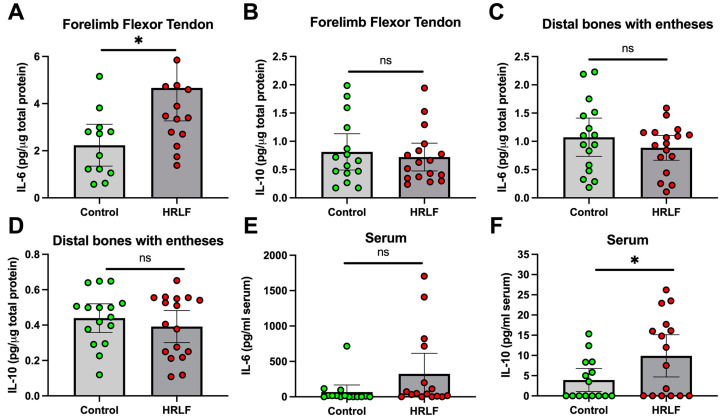
Pro/anti- or anti-inflammatory cytokine levels detected using ELISA. (**A**,**B**) IL-6 and IL-10 levels in forelimb flexor tendons. (**C**,**D**) IL-6 and IL-10 levels in distal bone with entheses. (**E**,**F**) IL-6 and IL-10 levels in serum. Musculoskeletal tissues were assayed in Control (*n* = 15–23) and HRLF (*n* = 13–18) animals; serum was assayed in control (*n* = 15) and HRLF (*n* = 16) animals. * *p* < 0.05, compared between groups as shown; ns = not significant.

The forelimb flexor muscles of the HRLF rats showed elevations in a cytokine that is both anti- and pro-inflammatory, IL-6, compared to the control rats [HRLF (0.53 ± 0.42 to 0.64 95% CI) versus control (0.36 ± 0.27 to 0.45 95% CI), *p* < 0.04] (Supplemental Figure S1D), but no differences in IL-10 [HRLF (0.08 ± 0.07 to 0.09 95% CI) versus the controls (0.09 ± 0.07 to 0.11 95% CI), *p* = 0.41] (Supplemental Figure S1E). In contrast, the flexor tendons of the HRLF rats showed elevated levels of IL-6 compared to the control rats (*p* < 0.05, Figure 3A), although no differences in IL-10 were found (Figure 3B). The distal forelimb bones with entheses in the HRLF rats showed no group differences in IL-6 and IL-10 (Figure 3C,D). In serum, there were no group differences in IL-6 levels (Figure 3E). However, circulating levels of IL-10 were elevated in the HRLF rats compared to the control rats (*p* < 0.05, Figure 3F).

### 2.3. Collagen Was Elevated and Muscle Reduced in Mature HRLF Forepaw Muscles

**Figure 4:**
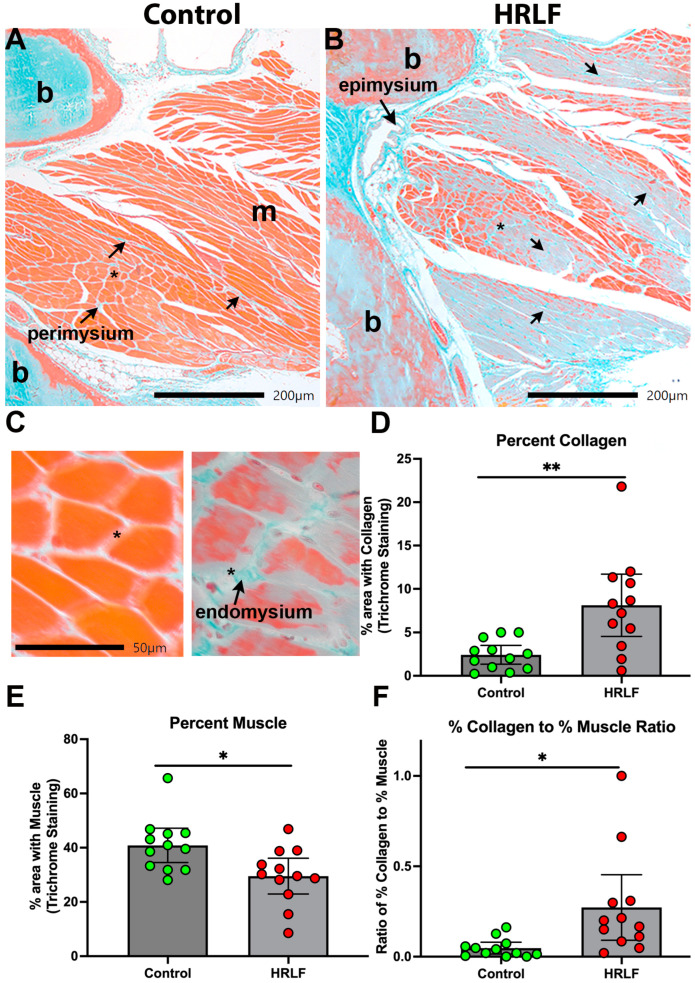
Collagen deposition and percentage of muscle in intrinsic hand muscles. (**A**,**B**) Representative example of interosseus muscles from control and HRLF groups after Masson’s trichrome staining (collagen is stained blue); b denotes bone, while m denotes interosseus muscle. In (**A**), arrows highlight perimysium examples. In (**B**), short arrows highlight areas of muscle with increased collagen deposition (fibrosis), longer arrows highlight the epimysium. (**C**) Higher (40×) magnification images from (**A**) on the left and (**B**) on the right (with endomysium indicated). Asterisks indicate the same region on the respective original image. (**D**,**E**) Graphs showing percent collagen staining and muscle staining in forepaw muscles. (**D**) Percent area of collagen. (**E**) Percent area of muscle. (**F**) Ratio of percentage of collagen to percentage of muscle. For all graphs, *n* = 12 per group. * *p* < 0.05 and ** *p* < 0.01, compared between groups as shown.

We next examined collagen deposition in histological sections of the intrinsic forepaw muscles using trichrome staining. The control rats showed typical amounts of blue collagen staining in perimysium slips between myofibers (Figure 4A). HRLF rats showed more collagen deposition within the perimysium and endomysial regions of the intrinsic forepaw muscles relative to the control rats, and what appeared either a loss of muscle or infiltration of collagen into the muscle fibers (Figure 4A–C). Quantification of the staining showed an elevation in the percent area with collagen staining (blue) in the HRLF rats compared to the control rats (*p* < 0.01, Figure 4D). In contrast, an analysis of muscle staining (red) in the same region of interest as assayed for collagen showed a reduction in percent area with muscle in the HRLF rats compared to the control rats (*p* < 0.05, Figure 4E). We compared the ratio of each for each replicate per rat, and observed a higher ratio of % collagen to % muscle in the HRLF rats than in the controls (*p* < 0.05, Figure 4F).

### 2.4. Evidence of Entheseal Histopathology in Mature HRLF Rats

**Figure 5:**
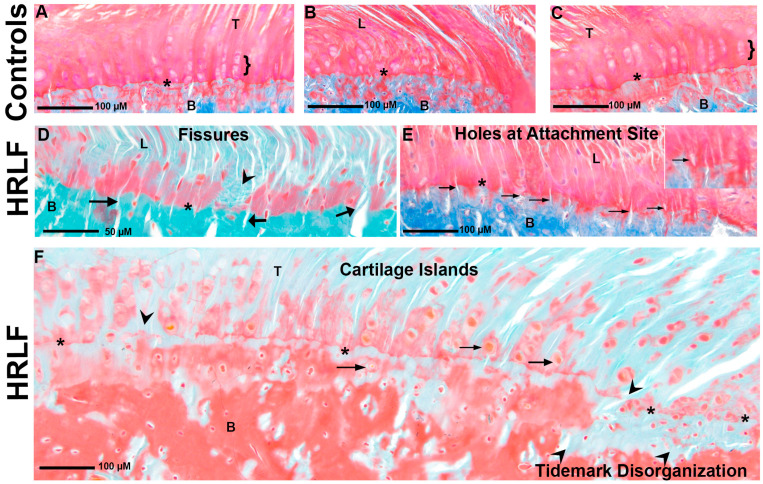
Entheseal pathology. Asterisks indicate the tidemark. (**A**–**C**) Representative examples of entheses from control animals. Images taken with a 20× objective after Masson’s Trichrome staining. Brackets indicate regions with typical cellular organization approaching the tidemark. Note the absence of such in HRLF examples (**D**–**F**). (**D**) Representative example of tidemark fissures (indicated by arrows) and tidemark disorganization (arrowhead). Image taken with a 40× objective after Goldner’s Trichrome staining. (**E**) Representative example of holes at the entheseal attachment site (indicated by arrows). Image taken with a 20× objective after Masson’s Trichrome staining. Enlarged example of a hole shown in inset in panel E (**F**) Representative example of cartilage islands (arrows) and tidemark disorganization (arrowhead). Image taken with a 40× objective after Goldner’s Trichrome staining. In all images, B = bone, T = tendon, and L = ligament.

Since the intrinsic forepaw muscles of the mature HRLF rats showed matrix changes, we hypothesized that the entheses would show cellular or matrix microdamage, as suggested previously (see methods for scoring methodology) [43]. Histological examples of entheses on carpal bone and the distal radius are shown in Figure 5. In control animals, we observed relatively continuous and smooth tidemarks. Small fissures and cartilage islands are occasionally seen in these mature control rats; however, most showed a typical tidemark shape and cellular morphology and organization (Figure 5A–C). In contrast, the entheses of the HRLF animals showed changes in structure, cellular organization, and integrity in the entheseal tidemark region (Figure 5D–F). There were increased mechanical interruptions along the tidemark boundary in the form of fissures (Figure 5D) and holes at the attachment site and fissures (Figure 5E and inset). There were notable increases in cartilage islands present near the tidemark (Figure 5F) and many distinct mechanical disruptions in the tidemark itself (indicated by arrowheads in Figure 5D,F).

**Figure 6:**
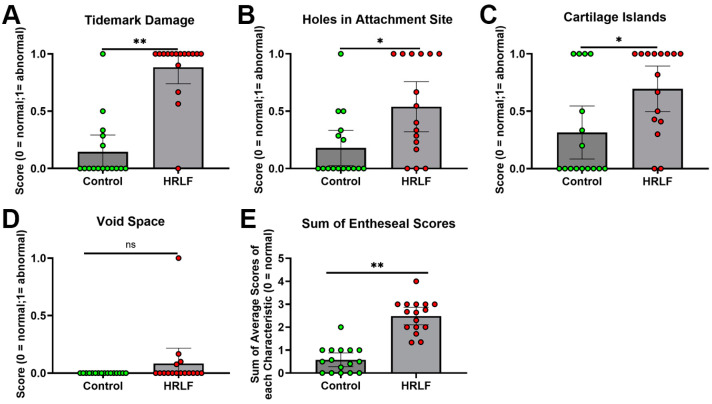
Quantification of entheseal pathology. (**A**) Tidemark disorder/damage was evaluated using a binary scoring system (see methods) with 1 indicating a loss of tidemark structure. (**B**) Holes in attachment zone were evaluated using a binary scoring system (see methods) with 1 indicative of tears or holes. (**C**) Cartilage islands were evaluated using a binary scoring system (see methods) with 1 indicating the presence of cartilage islands in the transition zone (**D**) Void space was evaluated using a binary scoring system (see methods) with 1 indicating the presence of void space in the entheses. (**E**) Sum of averages for each of the four domains of the grading scale. *n =* 16 in control group and *n* = 16 in HRLF group. * *p* < 0.05 and ** *p* < 0.01, compared between groups as shown; ns = not significant.

These changes were quantified using enthesis scoring of four domains: holes at attachment site, presence of cartilage islands within the fibrocartilage or bone, presence of fissures, and void spaces (Figure 6). Tidemark fissuring was higher in the HRLF group, compared to the control group (*p* < 0.01, Figure 6A). Also, in the HRLF rats, there were more holes in entheseal attachment sites (*p* < 0.01, Figure 6B) and more cartilage islands (*p* < 0.05, Figure 6C), compared to control rats. However, void space in the attachment zone did not differ between the groups (Figure 6E). The summed averages of entheseal changes were higher in the HRLF rats compared to the control rats (*p* < 0.01), indicative of enhanced entheseal microdamage in the HRLF group (Figure 6E).

### 2.5. Evidence of Radiocarpal Articular Cartilage Microdamage with HRLF Task

**Figure 7:**
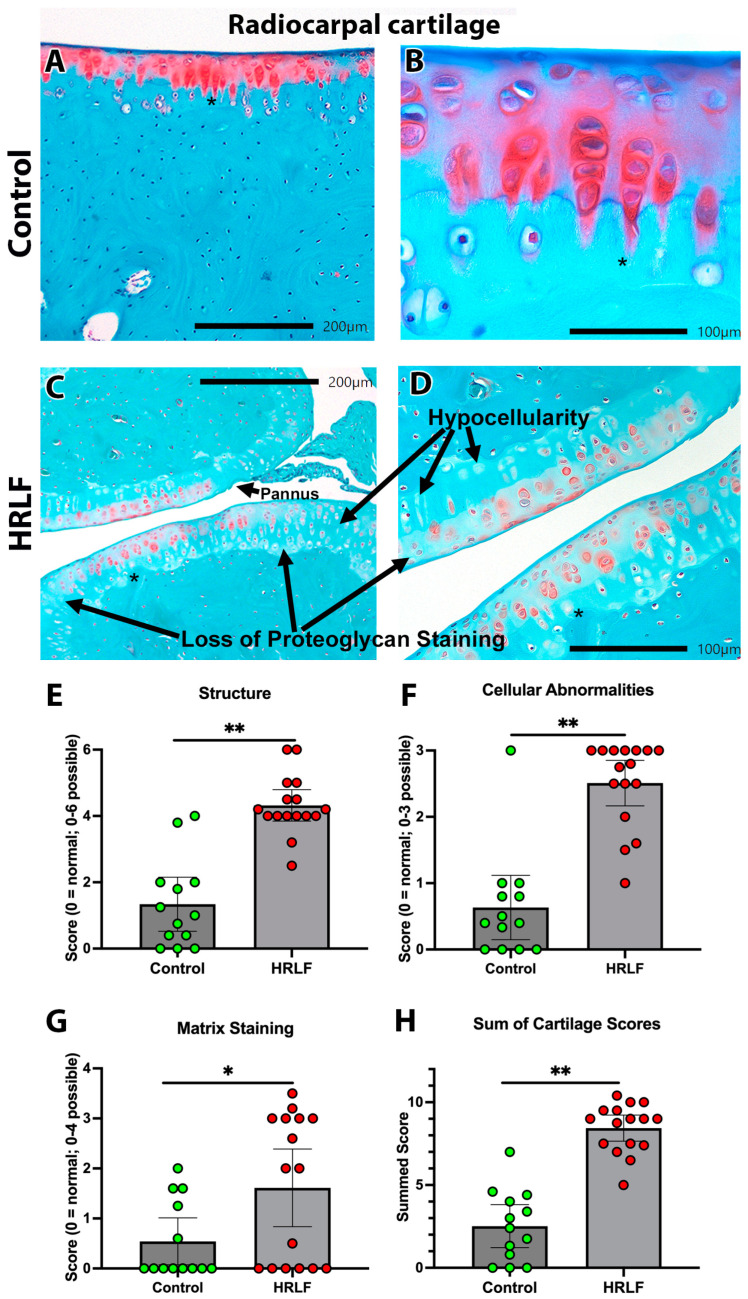
Radiocarpal cartilage pathology. Representative images of radiocarpal articular cartilage from each group after sections were stained with fast green and safranin orange. (**A**,**B**) Example from a control animal, depicting normal cellular organization (clear rows perpendicular to tidemark), normal articular surface, and matrix proteoglycan staining (red orange). Asterisks are placed in each paired row for orientation at higher magnification (**A** vs. **B**). (**C**,**D**) Examples from HRLF rats, showing reduced staining, hypocellularity and pannus. (**E**–**H**) Graphs showing quantification of pathology after scoring. (**E**) Structure scores for each group, with higher scores indicating more structural changes and/or deficits. (**F**) Cellular abnormality scores for each group, with higher scores indicating more cellular abnormality. (**G**) Matrix staining scores for each group, with higher scores indicating less proteoglycan staining in the articular cartilage. (**H**) Summed averages of (**E**,**F**,**G**), for each group. In (**E**–**H**), *n* = 13 for control group and *n* = 16 in HRLF group. * *p* < 0.05 and ** *p* < 0.01, compared between groups as shown.

Another key matrix structure undergoing loading in the wrist and forepaw during this repetitive lever-pulling task was the radiocarpal articular cartilage. Evidence of structural changes were visible in sections of radiocarpal articular cartilage from the HRLF rats (Figure 7C,D) relative to the control rats (Figure 7A,B). The HRLF group consistently showed more surface irregularities, hypocellularity, fissures, and pannus (defined as a rough and uneven cartilage surface) (Figure 7C,D). We scored the radiocarpal articular cartilage based on three characteristics: structure, cellularity, and matrix staining. The HRLF rats had higher scores for structural microdamage than the control rats (*p* < 0.01, Figure 7E), as well as higher scores for cellular abnormalities (e.g., hypocellularity) (*p* < 0.01, Figure 7F). The staining domain showed a significant reduction in proteoglycan staining (orange staining) in the HRLF rats (resulting in a higher pathology score) compared to the control rats (Figure 7G). The summed averages showed that the HRLF rats had higher overall scores for cartilage microdamage than the control rats (*p* < 0.01, Figure 7H).

### 2.6. Osteoclast Surface and Count at Trabecular Bone Below the Epiphyseal Plate

**Figure 8:**
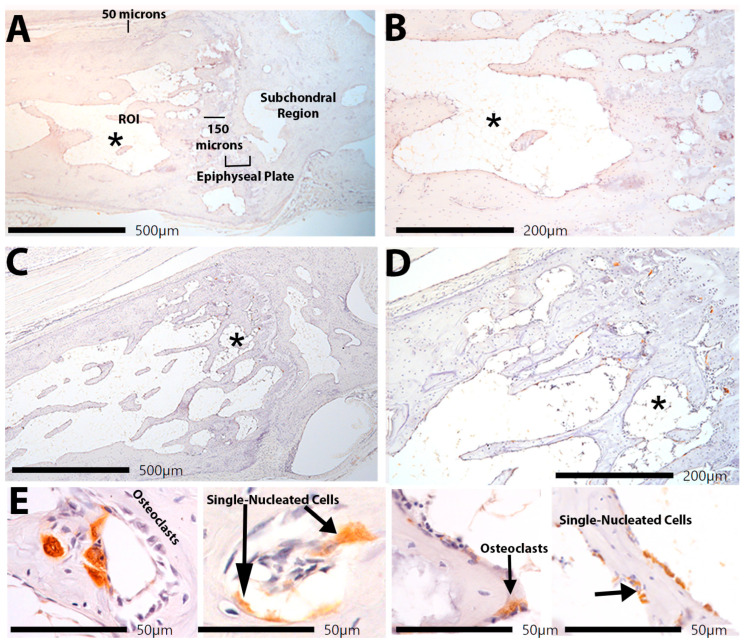
Distal radial metaphysis immunostained for CD68/ED1 (brown staining). (**A**–**E**) Representative example of this region in a control rat taken using a 4× (**A**) or 10× objective (**B**). (**A**) Distal forelimb region, with labels showing relevant landmarks. The epiphyseal plate and the subchondral region are noted. (**C**–**E**) Representative examples of this same region in HRLF rats taken using a 4× (**C**), 10× (**D**), or 40× (**E**) objectives. The asterisks in (**A**,**B**) and (**C**,**D**) are provided for orientation, as those pairs represent the same image at different magnifications. (**E**) Representative examples of multinuclear CD68/ED1+ cells (osteoclasts) and single-nucleated CD68/ED1+ cells (pre-osteoclasts or macrophages) with bone remodeling sites of metaphysis (left two images) and on the surfaces of trabeculae (right two images).

**Figure 9:**
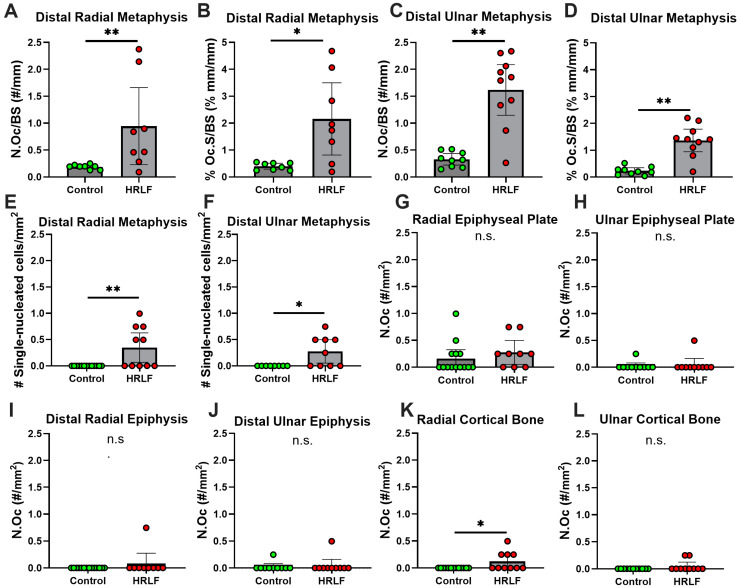
Quantification of CD68/ED1 immunostained multinucleated and single-nucleated cells in radial and ulnar bones, with distal metaphysis, epiphyseal plate, epiphysis, and mid cortical bone regions assayed. (**A**) Number of osteoclasts per bone surface (N.Oc/BS) in the distal radial metaphysis. (**B**) Osteoclast surface (Oc.S/BS, %) in the distal radial metaphysis. (**C**) Number of osteoclasts per bone surface (N.Oc/BS) in the distal ulnar metaphysis. (**D**) Osteoclast surface (Oc.S/BS, %) in the distal ulnar metaphysis. (**E**,**F**) Number of single-nucleated cells per millimeter squared in the distal radial and ulnar metaphysis, respectively. (**G**,**H**) Number of osteoclasts per millimeter squared in the radial and ulnar epiphysial plate, respectively. (**I**,**J**) Number of single-nucleated cells per millimeter squared in the distal radial and ulnar epiphyses, respectively. (**K**,**L**) Number of osteoclasts per millimeter squared on the mid cortical bone surfaces of radial and ulnar bones, respectively. *n* = 8 for both groups in (**A**,**B**). *n* = 9 for controls and *n* = 10 for HRLF in (**C**,**D**); *n* = 13–14 for Controls and *n* = 10 for HRLF in (**E**,**G**,**I**,**K**); and *n* = 10 for controls and *n* = 10 for HRLF in (**F**,**H**,**J**,**L**). * *p* < 0.05 and ** *p* < 0.01, compared between groups as shown; ns = not significant.

Since we have previously shown trabecular bone loss in mature rats performing the same HRLF task compared to both mature control rats and young adult rats performing the same HRLF task [25], we sought here to examine several regions of the distal forelimb bones for the presence of inflammatory cells that might contribute to bone matrix degradation. We immunostained bone sections with CD68/ED1, a marker that detects osteoclasts (multinucleated) and pre-osteoblasts or macrophages (single nucleated) and co-localizes with TRAP staining. The initial analyses of the trabecular region of the radial and ulnar metaphysis were performed in the metaphyseal trabecular bone (Figure 8A–C and Figure 9A–D), in accordance with published guidelines [44]. In the control animals, few to no multinucleated or single nucleated CD68/ED1+ cells were observed (Figure 8B). In HRLF animals (Figure 8C–E), we noted more multinucleated and single-nucleated CD68/ED1+ cells. Cell quantification is shown in Figure 9. In the radial and ulnar metaphyses, higher numbers of osteoclasts per bone surface were observed in the HRLF group, compared to controls (*p* < 0.01, Figure 9A,C). The osteoclast surface in these same regions was also higher in the HRLF group (*p* < 0.05 and *p* < 0.01, respectively, Figure 9B,D).

Regarding single-nucleated CD68/ED+ cells, we observed an elevation in these cells in the HRLF radial and ulnar metaphyseal regions of HRLF rats compared to controls (*p* < 0.01 and *p* < 0.05, Figure 9E,F, respectively). We performed additional analyses of osteoclast numbers and single-nucleated CD68/ED+ cells in the epiphysial plates, epiphyses, and mid-diaphyseal cortical bone of the radius and ulna (Figure 9G–L). Only the mid-diaphyseal cortical bone of the radius showed elevations in osteoclasts in the HRLF rats compared to the control rats (Figure 9K).

### 2.7. Grip Strength Correlated Inversely with Several Histopathological Changes

**Figure 10:**
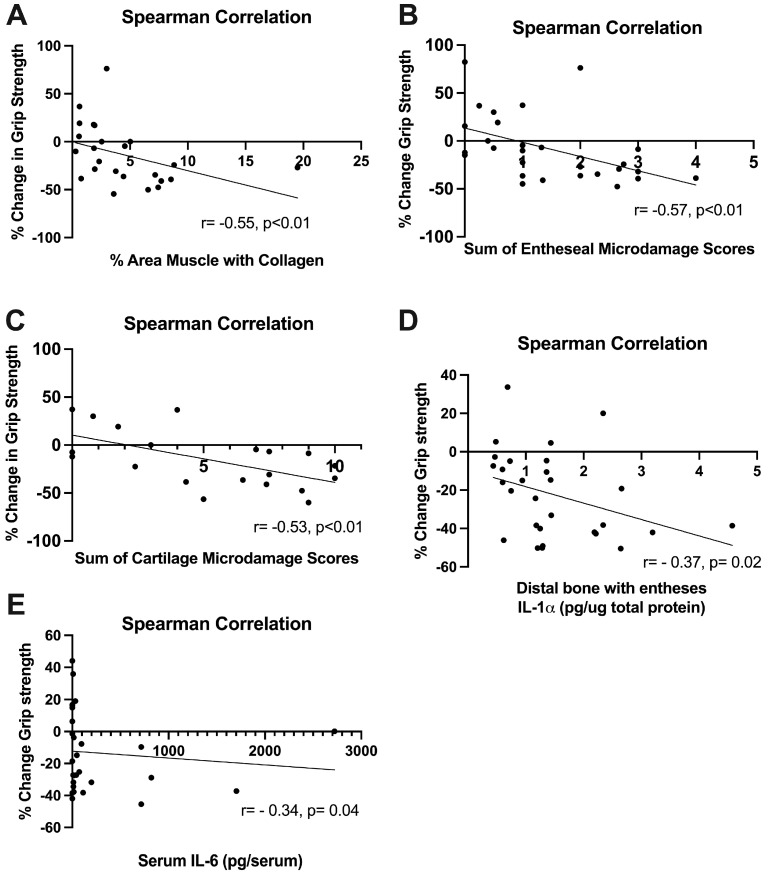
Grip strength changes and correlations with tissue outcomes. (**A**–**E**) Spearman’s r correlations for muscle, enthesis, cartilage, distal bone with entheses IL-1α, and serum IL-6, respectively. There was a moderate and significant negative correlation between collagen %, enthesis score, modified Mankin score (cartilage) and % change in grip strength, indicating that, as these metrics rose, there was a negative % change in grip strength. There was a weak and significant negative correlation between both distal bone IL-1α and serum IL-6 and % change in grip strength.

We have previously shown that declines in grip strength in rats following a task correlate with inflammatory changes (e.g., in [23]) and muscle fibrosis [31]. In this study, declines in grip strength correlated moderately with higher amounts of collagen in the intrinsic forepaw muscles assayed (Figure 10A), with higher scores for entheseal histopathology (Figure 10B), and higher scores for cartilage microdamage (Figure 10C). Declines in grip strength correlated weakly with higher levels of IL-1alpha in distal bone regions (Figure 10D) and higher serum levels of IL-6 (Figure 10E).

## 3. Discussion

We have previously established that a high-force paradigm creates marked changes in muscle, tendon, entheses cartilage, and bone [23,25,31]. However, overuse injury can occur from many different insults, and is not limited to only those performing high force tasks. Therefore, in this paper, we sought to comprehensively establish the changes that occur in the muscle–tendon–bone interface in the wrist and hand (forepaw in rats) in a low-force variation of our overuse injury model. Building on previous work noting bone changes in mature animals performing an HRLF task [23,25,45], here we report inflammatory and degradative changes in the muscle–tendon–bone unit and associated fascial and other connective tissues, such as entheses, as a consequence of task performance. We also report that many of these changes correlate with declines in grip strength.

Overuse-induced musculoskeletal disorders are varied in their etiology and diagnosis grouping, ranging from myositis to tendonitis, myalgias, and more [1,9,10,11,46]. Clinically, distal forearm pathologies such as wrist tendonitis and carpal tunnel syndrome are often seen [10,12,47]. Additionally, muscle fibrosis is often found in the aftermath of muscle strain or tear injuries in patients [48]. In most cases, a commonality appears to be repetition of load-bearing work over an extended period, leading to both local and systemic inflammation. Additionally, it is suggested that repeated overuse and strain can result in a ‘failed healing’ state in the tendon or enthesis which then lose function and structural integrity [49,50,51]. This theme of initial insult and inflammation leading to dysfunction and structural damage is echoed in our own findings, as discussed further below.

From a clinical standpoint, these observations and suggestions have been crucial in improving our understanding and contextualization of the results found in this study. With the 55+ age group being both the largest growing age demographic in the US and increases in delayed retirement, particular interest has risen regarding their workplace risks and needs [52]. While several socioeconomic factors contribute to the relationship between age and WRMSDs, it has also been noted that older workers tend to have higher severity of injuries compared to younger workers [53]. Even known socioeconomic factors, such as lifestyle (e.g., weight) and physical job demands, pose a greater risk of injury to older workers compared to younger workers [54]. An increased susceptibility to injury is thought to be a key contributor to these findings, and this is demonstrated through both our prior works and the present research [25]. Other work also notes a relationship between increased age and susceptibility to WRMSDs [12,47]. ‘Inflammaging’ may be contributory to this noted increase in susceptibility. A key feature of ‘inflammaging’ is increases in cellular senescence, which has also been noted in aging tendons and cartilage failure in osteoarthritis [55,56,57]. Interestingly, aging tendons show decreased metabolism, proliferation, and matrix secretion, and mature rats display higher rates of tendon fatigue failure [50,56]. Relatedly, age has also been shown to be a factor in intramuscular extracellular matrix (ECM) deposition [58,59,60]. Specifically, age-related alternations in intramuscular connective tissue, extracellular matrix mechanical properties, and alterations in hyaluronan and collagen in the extracellular matrices of muscle spindles were observed in these studies [58,59,60]. Those findings combined with our new findings highlight the increased basal deposition of ECM, and the reduced functionality of aged muscles. Additionally, these findings pair well with observations of the influence of inflammaging on tissue pathology [26,61,62], and contribute to an overall picture of the decreased adaptability and increased susceptibility that accompany aging.

We expanded on past studies examining cytokine levels in the serum, tendons, and forelimb bones (full length from elbow to wrist) of mature animals [23,24,25,42] to examine cytokine levels in forelimb muscles, distal forelimb tendons, distal bones at the level of the wrist with intact entheses, and serum. We observed tissue-specific variability in the pro- and anti-inflammatory cytokines examined. This was not unexpected, as past analyses by our lab have noted that changes in inflammatory cytokines are dependent on several factors, such as the age of the animal, systemic or local analyses, time of sampling, and duration and load of task [23,42,63]. Similar to past results in young animals carrying out a higher force task [22], TNF-α and IL1-α remained elevated in the bony tissues of the task animals, while tendons showed no increase in TNF-α at the 12 week task endpoint. This falls in line with previous literature that notes that chronic tendinopathy begins with an acute inflammatory phase, but eventually persists into a M2-macrophage-dominated failed and fibrotic healing response [64,65,66]. Additionally, we observed elevations of several inflammatory cytokines in the serum. Previous work regarding serum changes also shows us that systemic responses may peak earlier than the endpoint, with some pro-inflammatory cytokines peaking during shaping or after six weeks of tasks [23,67]. This later systemic response is a feature of our chronic overuse model [23,25,31,42,67]. Specifically, we saw increases in serum TNF-α, IL-10, MIP-1α, and CINC-2. TNF-α, MIP-1α, and CINC-2 are classic pro-inflammatory cytokines, generally thought to activate or recruit immune cells [68,69]. The increase in serum IL-10, a potent anti-inflammatory cytokine, is likely an attempt to shift towards proper homeostatic levels of immune activation [70]. These results align with prior reports in the field. A related reaching task in young rats performed for 6 weeks elicited changed behavioral changes in pain sensitivity, and yet no changes in muscle TNF-α or other examined cytokines [71]. A pinching model of median neuropathy in nonhuman primates also found significant dose-dependent neuropathic changes, despite no simultaneous increases in serum cytokines [72]. This previous work helps establish that while inflammatory changes may be present, their absence does not rule out functional declines. Here, we establish that the lasting structural changes, created by both mechanical and inflammatory influences, persist far beyond the resolution of inflammation and contribute to significant functional decline.

It is well established that chronic exposure to strain can lead to persistent fibrotic changes in muscles [73,74]. In our model, we have observed enhanced collagen deposition and production, and fibroblast proliferation, in muscles and various fascial tissues in younger rats (3–9 months of age) performing a high-repetition task for 3 to 18 weeks, with the greatest fibrotic responses observed at 18 weeks [28,75,76]. Fewer fibrogenic changes were observed in the musculotendinous tissues of rats performing a more moderate task (low-repetition high-force) for 12 weeks, and fewer still in rats performing a high-repetition negligible-force task for 6–9 weeks [77]. These exposure- and dose-dependent findings highlight the relevance of our present work, showing that just twelve weeks of a HRLF task in mature rats evoke fibrotic changes akin to 18 weeks of a more intense high-repetition high force task in younger rats. The increased susceptibility of mature rats to fibrosis after the performance of low-force tasks supports hypotheses indicating age as its own risk factor for the development of WRMSDs [5,53,78]. A loss of adaptive response may contribute to this increased susceptibility, with previous work noting the loss of adaptive myofiber hypertrophy response with age [79]. Said work proposes that the increase in interstitial tissue (fibrosis), blunted anabolic response, and decreased local stem cell reserves that come with aging all contribute to the reduced adaptive response in aging muscle [79]. It is also noted in other studies that chronic damage and inflammation contribute to shifting tissue fibrogenic changes [80,81]. This mechanism is supported in previous studies from our lab showing that anti-inflammatory drugs reduce the development of muscle fibrosis [31,77]. In these studies, we showed that anti-inflammatory drugs, such as ibuprofen, anti-TNF-α, and anti-CCN2, result in decreases in skeletal muscle fibrosis. Ibuprofen and anti-TNF-α were administered 3 weeks into the HRHF paradigm, while anti-CCN2 was administered after 18 weeks of HRHF tasks. As we have now established that fibrotic changes persist past the inflammatory cascade, we can see that it is possible to target fibrosis both during inflammation or and, depending on the drug target used.

The entheses studied in this experiment were of the fibrocartilaginous type, which are typically found attached to the epiphyses of long bones and on the small bones of the hands and feet [82]. Anatomically, fibrocartilaginous entheses are important for transmitting forces and providing cushioning in areas subject to high shearing and compressive forces [82,83]. This type of enthesis is typically characterized by four zones of tissue. Beginning with pure dense connective tissue, the enthesis transitions to an increasingly osteoid morphology. The dense connective tissue transitions to uncalcified fibrocartilage, to calcified fibrocartilage, and then to bone. Importantly, the area delineating the transition of uncalcified fibrocartilage to calcified fibrocartilage is referred to as the ‘tidemark’, and this area is subject to disorder and fissuring in several arthritic and inflammatory disorders [82,83,84,85,86]. The structural abnormalities we observed in the entheses of the forepaw and wrist could be a result of the elevated inflammatory cytokines (which are known to be cytotoxic), as well as damage from the repetitive loading in this region [87]. Persistent structural changes to entheses could be associated with longer term and further progression of dysfunction following an overuse injury [88]. Previously, we observed structural and cellular entheseal changes in the palm and wrist of young animals performing an HRHF task [31]. These changes correlated with functional declines that were not ameliorated by rest alone, indicating a persistence of the pathology even after the inflammatory cascade had long passed. We have additionally seen elevated cellularity in the wrist tendons of both young and mature animals performing an HRLF task [24]. Building on this, the present work identifies entheseal structural damage in mature animals performing an HRLF task. These findings correlated independently with functional declines and were present despite minimal local inflammation at the endpoint. We conclude that lasting and meaningful structural changes in the critical transitional zone of the muscle–bone–tendon unit remain after task.

In the field of sports medicine, it has been suggested that articular cartilage damage can persist into more chronic conditions after inflammatory changes exert their effect [89]. In present work, the cartilage of the mature animals performing our much lower force HRLF task was scored. In the scoring of cartilage changes, we noted elevated changes in the radiocarpal articular layer, including both large and small fissures, pannus, and disorders in the cellular layers. We observed varying degrees of abnormal cellularity and loss of matrix proteoglycan staining in our task animals. In other chronic musculoskeletal disorders, a commonly seen morphology involves the same surface fibrillation, chondrocyte clustering, and hypocellularity [75,90]. In osteoarthritic conditions, the chondrocytes in the articular cartilage first experience hypertrophy and increased functional demand. As the condition progresses these cells eventually become senescent or apoptotic and their function is lost [90]. This loss of function was evaluated by the matrix staining component of the Modified Mankin Scale. As the safranin-orange dye stains specifically for proteoglycans, a loss of staining indirectly shows missing chondrocyte function [91,92]. We saw the same spectrum of cellular abnormalities in the HRLF animals’ radiocarpal cartilage. The avascular nature of the cartilage leaves it uniquely susceptive to inflammatory changes [93]. Paired with the loss of reserve cells through senescence in aged joints, it is easy to see how the intersection of inflammation and already susceptible aging cartilage can lead to lasting structural deficits [62]. A knockout mouse model of OA, comparing the effects of age on the development of osteoarthritis, also noted a much greater sensitivity to injury and osteoarthritis development in aged animals [94].

We saw elevated osteoclast numbers per bone surface and osteoclast surfaces in both the ulna and radius of the task animals, matching past findings from this model [25,45]. We observed metaphyseal increases in both the ulna and radius of single-nucleated CD68/ED1 staining cells, which could be either preosteoclasts or single-nucleated monocyte lineage cells [95,96,97]. Paired with the cytokine changes, these single-nucleated cell changes contribute to the picture of pervasive inflammation and tissue damage seen in our mature animals. These findings align with previous findings we have noted, in which mature animals respond with a loss of bone integrity in the HRLF paradigm, whereas younger animals performing the HRLF task showed adaptive changes [25]. The increase in osteoclast numbers highlights inflammatory changes in the distal metaphysis, mirroring maladaptive changes present at every level of the distal forelimb muscle–tendon–bone unit. It was unsurprising to observe that the elevation in both single-nucleated cells and osteoclasts were contained mostly in the metaphysis. Several studies have noted that the influence of inflammatory and anti-inflammatory mediators differ based on the bone location [98,99].

Lastly, we evaluated changes in grip strength in all animals, finding significant negative correlations between all structural analyses and grip strength changes. Relating these observed histological changes to measured behavioral changes is important to establish that our histological data were not only significantly different between groups, but also related to a meaningful functional deficit. Grip strength is considered a mixed behavioral assay of lost muscle function and increased muscle pain [23,31]. In the literature, intramuscular injections of TNF-α result in declines in grip strength [100]. However, in our own model, we have found that declines in grip strength are not fully related to serum and muscle cytokine responses [23]. We found here that declines in grip strength correlated moderately with elevated muscle collagen, enthesis microdamage, and radiocarpal cartilage microdamage. Thus, we infer that the observed structural changes along the muscle–tendon–bone unit are functionally impactful and last beyond a potential acute inflammatory phase. This process has been observed in other work involving post-traumatic osteoarthritis, in which inflammation from an acute injury triggers lasting arthritic changes and pain in the affected joint [89,101].

There are limitations to mention. First, while we reported a decrease in the percent area with muscle in the HRLF group, these percentages were based on data from 3 to 8 muscle sections per forepaw, and per animal. While we included replicates to capture biological variability within the muscles, a more quantitative measure of collagen content might have been via ELISA or hydroxyproline assays. However, this was not possible in our study since we were examining intrinsic forepaw muscles (which are quite small), and we left the muscles intact with bones and tendons to assess enthesis morphology. Next, only female rats were included. This choice allowed us to directly compare our results to our past studies on aging female rats performing a similar task, enhancing our interpretation [23,24,25,42]. The force transducer sensitivity of our model setup is currently tailored to the pulling strength of female rats, so the inclusion of males may have reduced the quality of the data and made the interpretation of the findings more difficult and would have added sex as a potential confounder. Another reason for our focus on one sex is that human females have a higher incidence of work-related musculoskeletal disorders than males as WRMSDs affect females at a higher rate [1,102,103]. Current epidemiology work also suggests that WRMSDs in women are met with less support and have worse outcomes, making this an especially important demographic to focus on [104,105]. Additionally, existing work establishes that sex hormones, namely progesterone and estrogen, have profound effects on the tissues studied in this paper [106,107,108]. Estrogen receptors have been observed to be expressed in fibroblasts and chondrocytes, with roles in regulating the activity of these cells, as well as sensitization [106,107,108]. Finally, studies exploring differences in humans have shown increased joint laxity in females [109]—perhaps again due to the presence of female sex hormones. These established differences, as well as the historical underrepresentation of overuse injury research in females, strongly contributed to our rationale in utilizing a single-sex experimental paradigm. However, since human males also develop these disorders, future [110] studies are encouraged to include males.

## 4. Materials and Methods

### 4.1. Animals

All experiments were performed in accordance with the policies and recommendations of the Institutional Animal Care and Use Committee (IUCAC) of Temple University and in compliance with NIH guidelines for the humane care and use of laboratory animals. A total of 64 female Sprague Dawley rats (Charles River Laboratory, Wayne, PA, USA) were used (Figure 1A). Female rats were used to allow for a comparison to our previous studies [23,24,31,42] and because the examination of males, which are both larger and stronger, would require adjustments to the operant conditioning equipment and conditions, including a switch to higher-capacity force transducers as ours were chosen for their sensitivity to the force-generating capabilities of adult female rats. This change in equipment would confound our interpretation.

Female Sprague Dawley rats were procured between 9 and 11 months of age, and matured in house to 14 months of age. The study then began and continued until the rats were 18 mo of age. Rats were housed in an AAALAC-accredited central animal facility (under 12 h light–12 h dark cycle conditions, and with free access to water, 22–23 °C room temperature, cage changes 2/week, and filter-topped cages). Rats were group housed until the onset of the experiment. Thereafter, all rats were housed in individual transparent plastic cages due to the need for food restriction (described in the next paragraph). Rats were handled daily for at least 30 min, and provided with tunnels and ULAR (University Laboratory Animal Resources)-approved rat chew toys in their home cage for enrichment purposes. Sentinel rats housed in the same room were examined monthly for the presence of viral or bacterial infections as part of their regular veterinary care (no infections were detected).

Beginning at 14 mo of age, the rats were food-restricted to within 95% of their naïve body weight to motivate them to participate in the operant task (HRLF and control rats were similarly food restricted). All animals were weighed weekly and their food was adjusted to maintain that percentage of naïve body weight (i.e., no more than 5% weight loss). At this point, the rats were randomly divided into control (*n* = 32) and high-repetition low-force (HRLF, *n* = 33) groups (Figure 1A).

### 4.2. Repetitive Overuse Task (High Repetition Low Force Task)

The lever-pulling apparatus used was as previously described [45] and as shown in Figure 1B. A shaping period was conducted across four weeks as previously described [25], with the goal of shaping the correct pulling behavior in the HRLF animals. The required pulling force for this low-force task was increased stepwise across each week of the shaping period until the target pull force of 15% of their mean maximal pulling force (29 cN) was achieved. There was no specified reach rate during this shaping period. The shaping occurred for 15–20 min/day, 5 days/week, for 4 weeks.

These animals went on to perform the HRLF task for 12 weeks (Figure 1A) at a target reach rate of 4 reaches/min, at a target force of 15 ± 5% of their mean maximum pulling force (26.4 ± 1.32 cN), in four 30 min sessions, with 1.5 h of rest between each session, for 3 days/wk. The rats were expected to hold the lever bar at the target force for at least 500 msec, once every 15 s. Since this is an operant task, the rats were free to pull at other times or force amounts. Additionally, rats were free to use either arm, or both, with their daily preference for right (R), left (L), or both (B) being noted by observers. Only data from the primary reach limb are reported to improve interpretation. However, if the animals reached outside of the cueing period, or over- or under-reached, no food reward was delivered, as depicted previously [25,67]. The ForceLever software (version 4.3, release 1, build 58) tracked the number of successful and unsuccessful reaches, and reported them automatically at the end of each session. Successful pulls were defined as pulls made within the correct time frame (once every 15 s when an auditory indicator was clicking), within the target force range (26.4 ± 1.32 cN), and with the lever bar held at that force for at least 500 msec. These pulls were tracked in each session and used analyzed to determine animal learning and task success (Figure 1D). These data are only present and analyzed for animals that performed the task (only task rats versus control rats). Limb use in the HRLF rats was tracked during each session and showed that most of the rats used both limbs to reach. Shaping and task sessions were performed on the same day each week and at the same time each day, when allowed by holiday scheduling. Animals with a noted limb preference during task performance had the non-dominant limb excluded in any subsequent analysis. If both limbs were used similarly, data from each forelimb with attached forepaw (right and left) were graded and combined.

### 4.3. Grip Strength Behavioral Assay

Reflexive grip strength was measured in all rats in the study bilaterally using a commercially available rodent grip strength meter (Stoelting, Wood Dale, IL, USA). This was performed by gently elevating the rats by the tail until they grasped the meter with the preferred limb. The animal was then pulled gently and continuously away from the bar until their grip was released. The instrument recorded the maximum force of the pull at the time of the release in grams of force, which was converted to cN. The grip strength assay was performed once before the shaping period began (baseline) and at the conclusion of the experiment (post-test). The percent change in grip strength in preferred reach limbs, from baseline levels, was then calculated per rat, as previously described [28]. Preferred limb was determined by a consistent pattern of limb preference throughout the experiment, noted by the personnel running the reach task.

### 4.4. Tissue Collection

After the 12-week HRLF task was completed, all animals were euthanized for tissue collection. At this time, the control (*n* = 32) and HRLF (*n* = 33) groups were further subdivided for technical reasons into animals used for either biochemical or histological analyses: control rats, biochemical (*n* = 16) and histological (*n* = 16); HRLF rats, biochemical (*n* = 17) and histological (*n* = 16).

For this, at 18 h after completion of the final task session (to avoid acute increases in serum cytokines associated with task performance), animals were deeply anesthetized with 5% isoflurane in oxygen. Depth of anesthesia was assessed and monitored by the pattern and rate of respiration, the absence of muscle tone, and the absence of toe and tail pinch, and eye blink reflexes. When the animals no longer showed any reflexive responses, an absence of muscle tone, and breathing had halted, the animals underwent a thoracotomy and cardiac puncture. Blood was collected from all animals by cardiac puncture using an 18-gauge needle and centrifuged, and the supernatant was collected as serum and stored at −80 °C for later use in ELISAs.

For animals from which tissues were collected for biochemical analyses, after prior thoracotomy and cardiac puncture for blood draw, forearm flexor muscles (which included primarily the flexor digitorum muscle yet also the flexor carpi radialis and ulnaris), forearm digitorum tendons extending from the forearm muscle mass into the forepaw, distal forelimb bones (radius and ulna) with their associated entheses left intact, were dissected out bilaterally, and collected as flash-frozen tissues and stored at −80 °C for later use in ELISAs.

The remaining animals were prepared for histological analyses. After thoracotomy and cardiac puncture for blood draw, intracardial perfusion was performed, first with saline and then with 4% buffered paraformaldehyde. Forelimbs with attached forepaws were dissected out and fixed by immersion overnight in buffered 4% paraformaldehyde before being processed for plastic or paraffin embedding (19 animals and 25 animals, respectively). For plastic embedding, forelimb/forepaw bones were embedded in methyl methacrylate resin (17734-1, MMA, Osteo-Bed Bone Embedding Kit, Polysciences, Warrington, PA, USA). For paraffin embedding, forelimb/forepaw bones were decalcified in Immunocal (1414-1, McKinney, TX, USA) before paraffin embedding. Forepaw and distal forelimb bones were sectioned into 5 um longitudinal sections and placed on charged slides. Enough sections on slides were prepared such that there were at least 3 replicates for each animal and limb side.

### 4.5. Biochemical Assays

ELISAs were used to examine serum levels of IL-1α, TNF-α, MIP-1α/CCL3, CINC-2/CXCL3, IL-6, and IL-10 in control (*n* = 7–15) and HRLF (*n* = 9–19) animals. Blood was collected, as described above. Samples were chilled for 1 h following collection, centrifuged at 1500 rpm for 20 min, and serum supernatant was collected, which was frozen immediately until use. Samples were thawed on ice and assayed using a commercial multiplex ELISA system, using previously described methods and sources [23,67]. Results are reported as pg/mL serum. Single-plex ELISAs were used to assay forelimb muscle, forelimb/forepaw tendons, and distal bone with entheses for IL-1α, TNF-α, IL-6, and IL-10, using commercially available ELISA kits for rat tissues (BioSourceTM, Carlsbad, CA, USA) in control (*n* = 15–23) and HRLF (*n* = 13–19) animals. Tissue ELISA data were normalized to total protein concentration as measured using BCA protein assays (23227, ThermoFisher Scientific, Rockford, IL, USA), and then presented as pg/microgram of total protein.

### 4.6. Histological Staining and Analyses

Slides were deparaffinized or deplasticized using xylene, with those in plastic requiring overnight xylene treatment in a 37 °C oven for complete deplasticization. Subsets of sections on slides underwent either trichome (Masson’s or Goldner’s) or fast green/safranin-orange staining. Using both stains has been suggested for a better quality of detail in different tissue regions as well as to ensure consistency of observations [111]. Thereafter, sections were dehydrated in increasing concentrations of ethyl alcohol, cleared in xylene, and cover-slipped with DPX (0652, Sigma-Aldrich, St. Louis, MO, USA) as the mounting medium. Masson’s or Goldner’s trichrome-stained slides were used to examine collagen (stains blue after trichrome staining) and muscle tissue (stains reddish after trichome staining) visualization. For all analysis, the individual carrying out the analysis was naïve to group assignment.

The quantification of collagen versus muscle profiles was performed in trichrome-stained sections from control (*n* = 12) and HRLF (*n* = 12) rats using an image analysis system: an E800 Nikon microscope (Nikon, Melville, NY, USA) with a digital camera (Jenoptik Gryphax, Sanford, NC, USA) integrated with a quantification software (Bioquant Osteo 2022 Version 22.5.60 MP) on a Windows 11 PC computer. A pixel count threshold was used in which the number of blue pixels (collagen) or red pixels (muscle) in the same irregular region of interest (the area containing the muscle and associated fascial tissues, i.e., perimysium and endomysium) were counted by the software, as were the total number of pixels in that same region of interest, using previously described methods [112]. The area being quantified was selected to avoid osteoid and tendonous tissue and the irregular interest selection tool of the software. The percentage area with collagen staining was calculated as the number of collagen pixels in a region of interest, divided by the total number of pixels in the region of interest, multiplied by 100. The percentage of muscle was similarly calculated. At least 3 different areas were scored per animal. The average area examined included 1.69 mm^2^.

The trichrome-stained slides were also used for the enthesis analyses (*n* = 16 per group). Enthesis scoring was performed by binarily scoring four domains: (1) holes at the attachment site, (2) cartilage islands, (3) tidemark fissuring, (4) void space, or (5) vascularization of the enthesis. Briefly, holes at the attachment site were indicated by non-artifact gaps where the enthesis joined bone. Cartilage islands were marked by inappropriate cartilage deposits in the transition zone or the bone. Tidemark fissuring or disorder were marked by tears in the tidemark (discontinuity) or loss of normal curvature. Void spaces were marked by regions in deeper layers of the enthesis with missing tissue. Finally, vascular invasion was not often noted, but we scored the presence of blood vessels in any of the entheseal layers. A score of 0 indicated no presence, while a score of 1 indicated the presence of the said morphological change. Each animal limb was graded using available replicates, with the reported score being an average of the subdomain score for all replicates. The averages of each domain’s score per distinct rat and limb were then summed to give a “Summed Average” score.

Slides stained using the fast green/safranin orange protocol were used to examine articular cartilage changes in control (*n* = 13) and HRLF (*n* = 16) animals, after being deparaffinized or deplasticized. After staining, these slides were then dehydrated in ethanol, cleared in xylene, and cover-slipped using DPX. The medial radiocarpal area was assessed for morphological changes using a modified Mankin scoring system, as previously described [29]. A 3-domain scale assessing structure, cellularity, and staining was used. Within the structure domain there were 7 possible scores: 0 = normal; 1 = irregular surface (including fissures); 2 = pannus; 3 = superficial cartilage layers absent; 4 = slight disorganization (absence of cellular rows; presence of small clusters); 5 = fissures into calcified cartilage layers; 6 = gross disorganization (>25% of joint with chaotic structure, clusters, and osteoclast activity). Within the cellularity subdomain there were 4 possible scores: 0 = normal; 1 = hypocellularity; 2 = clustered; 3 = hypocellularity. The staining domain had 5 possible scores: 0 = normal or slight reduction in staining; 1 = staining reduced in radial layer; 2 = reduced in interterritorial layers; 3 = only present in pericellular matrix; 4 = staining absent. Replicates’ scores per rat were averaged on each subdomain for each limb of each rat. The averages of each domain’s score per distinct rat and limb were then summed to give a “Summed Average” score.

Distal forelimb and forepaw bones with attached entheses were analyzed in control rats (*n* = 8–14) and in HRLF rats (*n* = 8–10) for CD68 multinucleated and single-nucleated cells immunohistochemically using a specific CD68 [ED1] antibody (ab31630, Waltham, MA, USA) previously shown to co-localize with Tartrate-resistant acid phosphatase isoform 5b (TRAP5b). After counterstaining with hematoxylin, stained sections were analyzed using a Nikon E800 epifluorescent microscope (Nikon, Melville, NY, USA) with a customized X-Y motorized stage (Applied Scientific Instruments, Eugene, OR, USA) and a digital camera (Gryphax Jenoptik, Jena, Germany). For analysis of the metaphysis, the region of interest was selected to be 150 microns proximal to the epiphyseal growth plate and 50 microns inward from the cortical bone. Imaging software (Bioquant Osteo 2022 Version 22.5.60 MP) was used to perform the analysis and quantification. Multinucleated osteoclasts were quantified according to the recommendations of the American Society for Bone and Mineral Research [44]. Numbers of osteoclasts (multinucleated CD68+ cells) per bone surface (N.Oc/BS, #/mm) and osteoclast surface (% Oc.S/B, % mm/mm) were counted in stained sections on trabecular surfaces in the secondary spongiosa, as were the number of single-nucleated CD68+ cells in these same regions, with the results presented as # CD68+ cells (#/mm). Numbers of osteoclasts (multinucleated CD68+ cells) were quantified as # CD68+ cells (#/mm) in the mid-diaphyseal cortical bone of the radius, and epiphyseal plate and epiphysis of both the radius and ulna.

### 4.7. Statistical Analysis

The sample size for this study was derived from our previous studies involving mature rats [24,42]. In those studies, the number of mature animals analyzed per group were *n* = 4–18 (with a mean of *n* = 10) for behaviors [42], *n* = 6–7 for serum cytokines, and *n* = 3–7 (with a mean of *n* = 5) for tissue cytokines [24,42]. From these data, a power analyses set at conservative thresholds of 80% power and a 0.05 alpha level indicate that at least 7 rats/group were needed for histological and biochemical analyses. Therefore, in this study, similar or more animals per group were analyzed: *n* = 7–21 for biochemical analyses, and *n* = 8–16 for histological analyses, with exact number indicated in the scatter plot bar graphs.

Data were first tested for normality. The Shapiro–Wilk test was used when the data set allowed, with the Kolmogorov–Smirnov test being used otherwise. If the data had a normal distribution in both groups, they were analyzed using a parametric *t* test with Welch’s correction assuming equal SDs. If normality was unable to be assumed, it was analyzed using a non-parametric comparison [113]. For ranked data (such as our entheseal scores and cartilage scores), a Mann–Whitney test was used. In other data with tied values (for example, osteoclast count in which there were many observations of zero), a Mann–Whitney test was also used. For all other continuous data, a Kolmogorov–Smirnov test was performed. Each data point from the same rat and limb side was grouped as a replicate. Percentage change in grip strength data were correlated with percent area muscle with collagen, and sum of entheseal scores, sum of cartilage scores, and cytokines in tissues and serum, using Spearman’s r correlation. Values between ±0.3 and ±0.59 were interpreted as a moderately positive or negative relationship, between ±0.6 and ±0.79 as strong relationships, and between ±0.8 and 1.0 as very strong correlations [114,115]. However, these values are arbitrary limits and the correlation results should be considered in context.

## 5. Conclusions

In this work, we characterized the changes that can happen during an overuse injury and showed that aging populations may have an increased susceptibility to such injuries. Previously, we showed that mature rats in overuse models are more susceptible to radial bone loss, elevated fibrotic protein changes, inflammatory changes in the tendons, and behavioral displays of lost function and increased pain [22,24,25,28]. In the current study, we expanded on these findings by showing changes along the entire muscle–tendon–bone unit, including the enthesis, and performed an analysis of cytokine changes at affected tissues and systemic changes. These data demonstrate the inflammatory and degradative changes in the forearm/forepaw tissues of mature female animals exposed to prolonged repetitive tasks, changes with clinical relevance for work-related overuse injuries in mature human females.

## Data Availability

The raw data supporting the conclusions of this article will be made available by the authors on request.

## Supplementary Materials

The following supporting information can be downloaded at: https://www.mdpi.com/article/10.3390/ijms252413546/s1.
