# Supplementary material for: Repetitive Overuse Injury Causes Entheseal Damage and Palmar Muscle Fibrosis in Older Rats
Source: Int J Mol Sci. 2024 Dec 18;25(24):13546. doi: 10.3390/ijms252413546 (PMC11679654; doi:10.3390/ijms252413546)
Supplement: Supplementary file 1 [file ijms-25-13546-s001.zip › ijms-3355369-supplementary.pdf]

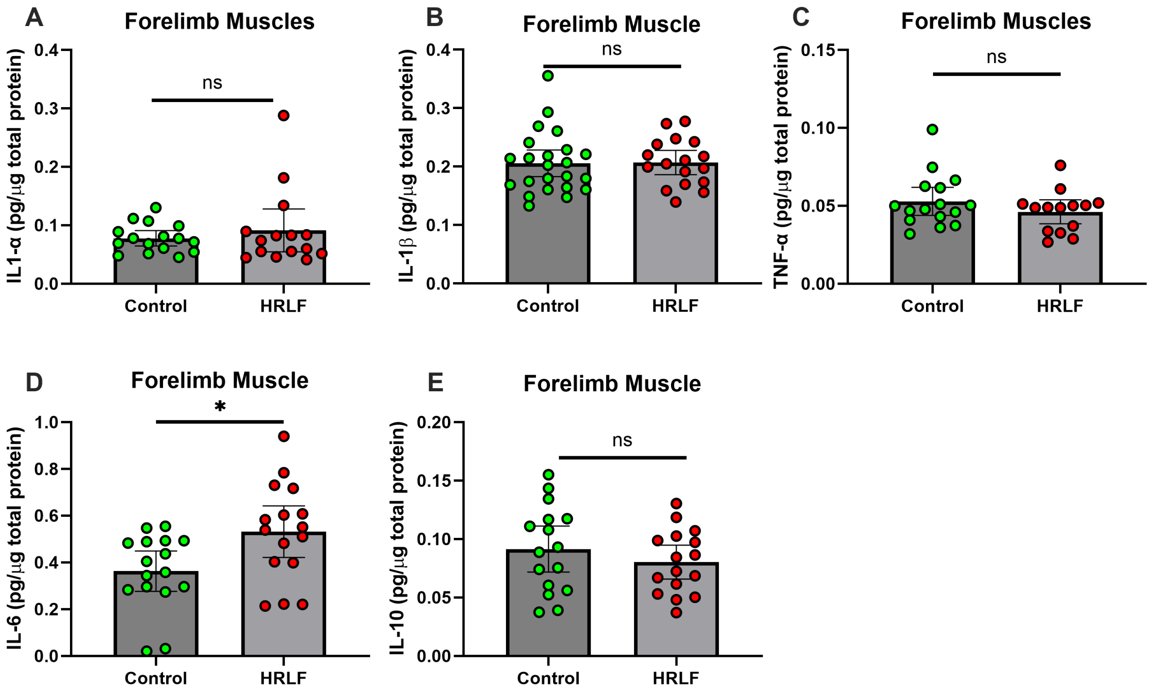

Supplemental Figure S1. Shows forelimb muscle cytokine data, gathered via ELISA. (A) IL-1α data compared between Control and HRLF groups. (B) IL-1β data compared between Control and HRLF groups. (C) TNF-α data compared between Control and HRLF groups. (D) IL-6 data compared between Control and HRLF groups. (E) IL-10 data compared between Control and HRLF groups. N=13-23 for Control groups and N= 13-17 for HRLF groups. * indicates significance at the 0.05 level.
